# The Phenomena and Criteria Determining the Cracking Susceptibility of Repair Padding Welds of the Inconel 713C Nickel Alloy

**DOI:** 10.3390/ma15020634

**Published:** 2022-01-14

**Authors:** Katarzyna Łyczkowska, Janusz Adamiec

**Affiliations:** Faculty of Materials Engineering, Silesian University of Technology, ul. Krasińskiego 8, 40-019 Katowice, Poland; janusz.adamiec@polsl.pl

**Keywords:** high-temperature brittleness range, hot cracking, TIG welding, transvarestraint test, Inconel 713C, nickel alloy

## Abstract

The creep-resistant casting nickel alloys (e.g., Inconel 713C) belong to the group of difficult-to-weld materials that are using for precise element production; e.g., aircraft engines. In precision castings composed of these alloys, some surface defects can be observed, especially in the form of surface discontinuities. These defects disqualify the castings for use. In this paper, the results of technological tests of remelting and surfacing by the Tungsten Inert Gas method (TIG) in an argon shield and TecLine 8910 gas mixture are presented for stationary parts of aircraft engines cast from Inconel 713C alloy. Based on the results of metallographic studies, it was found that the main problem during remelting and pad welding of Inconel 713C castings was the appearance of hot microcracks. This type of defect was initiated in the partial melting zone, and propagated to the heat affected zone (HAZ) subsequently. The transvarestraint test was performed to determine the hot-cracking criteria. The results of these tests indicated that under the conditions of variable deformation during the remelting and pad welding process, the high-temperature brittleness range (HTBR) was equal 246 °C, and it was between 1053 °C and 1299 °C. In this range, the Inconel 713C was prone to hot cracking. The maximum deformation for which the material was resistant to hot cracking was equal to 0.3%. The critical strain speed (CSS) of 1.71 1/s, and the critical strain rate for temperature drop (CST), which in this case was 0.0055 1/°C, should be used as a criteria for assessing the tendency for hot cracking of the Inconel 713C alloy in the HTBR. The developed technological guidelines and hot-cracking criteria can be used to repair Inconel 713C precision castings or modify their surfaces using welding processes.

## 1. Introduction

Nickel-based casting alloys are widely used; e.g., in the aviation industry as materials for engine elements such as high- and low-pressure turbine blades, control segments, etc. [1,2,3]. Such components are manufactured by precision casting, which enables castings of a high dimensional accuracy and with the correct shape to be obtained without the need for further mechanical treatment. Analysis of the literature data indicated a considerable proportion of castings are disqualified for use due to identified casting defects in the form of pores, blowholes, shrinkage porosities, or cracks [4,5]. In the industry, these types of defects are commonly repaired by welding techniques.

Analysis of the present knowledge on weldability of nickel-based casting alloys indicated that the main limitation of the repair and remanufacturing of such precision castings is the hot-cracking effect. Hot cracks most often run along the weld/pad weld axis, or as intercrystalline cracks [6].

**Figure 1:**
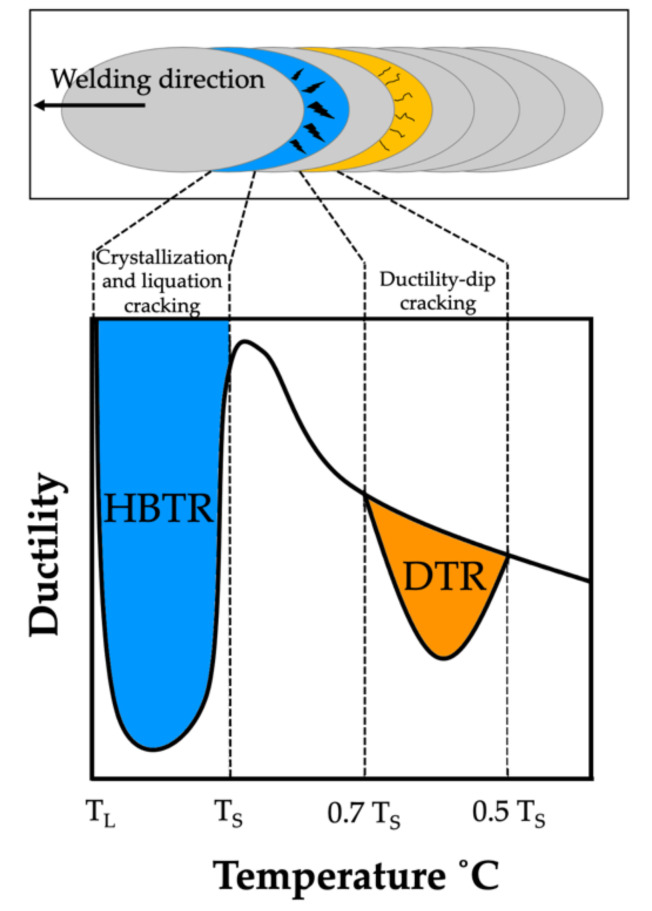
Areas of hot-crack initiation in the weld and the heat affected zone.

**Figure 2:**
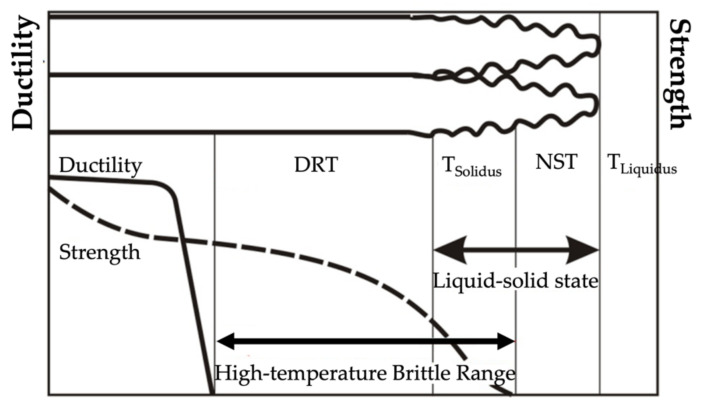
The high-temperature brittleness range (HTBR) determining crystallisation cracking in welded joints and padding welds. DRT—ductility recovery temperature; NST—nil strength temperature [16].

The authors of [7,8,9,10,11] pointed out that the most common cause of low resistance to hot cracking was plastic deformation in the material during weld crystallisation, leading to the rupture of the liquid film along dendrite boundaries, as well as the deformation growth rate and the temperature brittleness range. Cracks the form during welding (crystallisation and liquation cracks) initiate within the high-temperature brittleness range (HTBR), whereas cracks occurring below the solidus temperature—ductility-dip cracking (DDC) cracks (Figure 1)—are related to the ductility-dip temperature range (DTR) [6,12,13,14,15]. The HTBR is defined as the range between the nil strength temperature (NST) upon heating and the ductility recovery temperature (DRT) upon cooling [16,17]. The types of hot cracks that form in the HTBR or the DTR depending on the welding temperature are shown in Figure 2. 

The most frequently occurring type of hot crack is the crystallisation crack. During the final phase of crystallisation, nickel-based alloys display a tendency towards the segregation of alloying elements along the solidification grain boundary, which leads to the formation of a liquid film. The liquid film is characterised by poor mechanical properties and ruptures as a result of local tensile stresses related to weld shrinkage, which in turn leads to the initiation of a crack [18].

**Figure 3:**
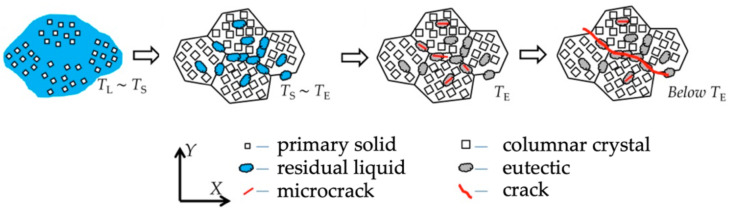
Schematic diagram of the crystallisation cracking mechanism.

The number of crystallisation cracks depends i.a. on the number and nature of intermetallic phases formed during solidification, the surface tension of the liquid metal, the distribution of the liquid at the final phase of crystallisation, the solidification temperature range, the weld’s tendency towards shrinkage, etc. The process of crystallisation cracking is presented in Figure 3.

According to the theory described by J. F. Lancaster in [19], materials having a wide HTBR are characterised by a low strength/temperature gradient, and thus are susceptible to crystallisation cracking, whereas materials with a narrow HTBR are resistant to crystallisation cracking. The work also demonstrated that a major factor affecting the crystallisation cracking susceptibility of a material is its ductility. The higher the ductility, the better the cracking resistance [19].

**Figure 4:**
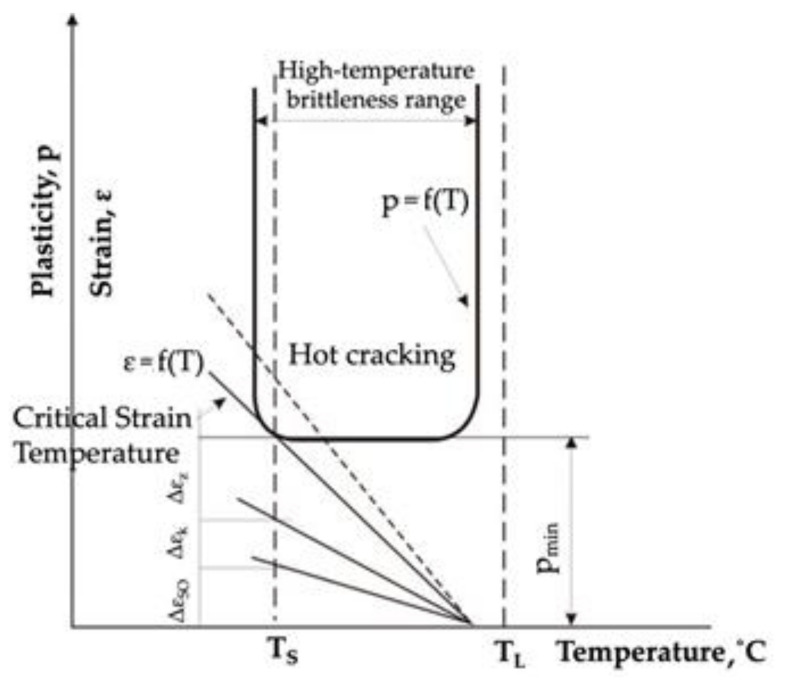
Dependence of alloy ductility within the HTBR and the strain rate [20].

However, the basic theory describing crystallisation cracking within the high-temperature brittleness range was presented by N.N. Prokhorov [20]. In his work, he assumed that there was a certain reserve of material plasticity (A = CST·HTBR (%), being the product of the HTBR width (°C) and a parameter referred to as the critical strain rate for temperature drop (CST) (%/°C). He claimed that the main measure of crystallisation-cracking susceptibility was the material’s plasticity within the HTBR (Figure 4).

During weld crystallisation, weld ductility drops to a value referred to as p_min_. Cracking occurs if the built-up strain during weld crystallisation exceeds the HTBR; accordingly, if the accumulated strain related to free shrinkage and the change in the weld shape is lower than p_min_ and falls within the reserve of plasticity, no cracking will occur in the welded joint. 

The research results published in [21,22,23,24,25], concerning crystallisation cracking in nickel-based casting alloys, also confirmed that such cracking was caused by the contamination of the material with low-melting phases. During weld crystallisation, they segregated towards grain boundaries, and thus reduced the material’s ductility within the HTBR. It was found that the materials described had a wide HTBR, which resulted in crystallisation cracking. 

**Figure 5:**
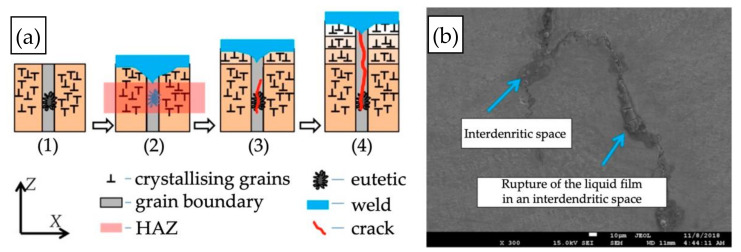
Liquation cracking mechanism: (**a**) diagram; (**b**) liquation cracks between dendrites [6].

A second type of hot crack is the liquation crack, which forms most frequently in nickel-based alloys with a high Al + Ti content. The literature points to the presence of the γ′ phase—Ni_3_(Al, Ti)—as their main cause [6,26]. They form due to the recrystallisation of low-melting eutectic mixtures based on partially melted γ′ phase, which leads to the formation of a thin liquid film along dendrite boundaries. Such cracks are usually identified along grain boundaries within the partially melted zone [14]. Elements such as B, S, and P, which segregate towards grain boundaries, also contribute to higher susceptibility to such cracks [11]. The mechanism of liquation crack formation is shown in Figure 5.

Cracks of this type have been described in the literature mainly with regard to austenitic steels and a number of nickel-based alloys; however, there are no precise and exhaustive descriptions of the liquation-cracking mechanism in welded joints and padding welds of nickel-based casting alloys, including Inconel 713C. 

**Figure 6:**
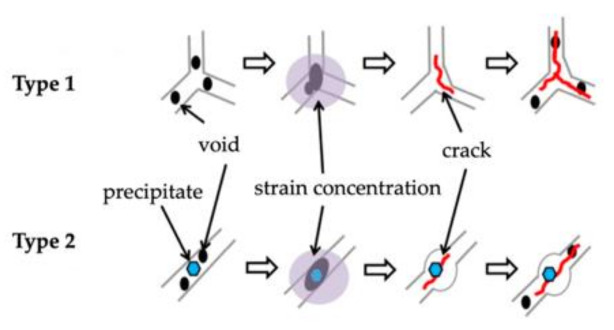
Schematic diagram of the ductility dip cracking mechanism.

A third type of hot crack is the DDC crack. Such cracks occur within 0.5 ÷ 0.7 of the solidus temperature; i.e., within the ductility-dip temperature range (DTR) in the solid state. It is deemed that the main cause of this type of cracking is the formation of microvoids along the boundaries of crystallising grains (Figure 6, Type 1) or the partial melting of carbides (Figure 6, Type 2), as well as thermal stresses during crystallisation and low metal ductility within the DTR. This leads to plastic deformations in the material, which depend i.a. on the material’s thermal conductivity, the crystallisation rate, the presence of impurities in the welded joint, and interdendritic microporosity. If the strain exceeds the limit values, cracks will initiate in the material [6].

Published articles have described the DDC phenomenon mainly for wrought nickel-based alloys; e.g., Alloy 690 [27], Inconel 625, and Inconel 600 [28]. Some works on casting alloys such as Inconel 738 [29] have also been published. The research indicated that the main cause of such cracking was the partial melting of carbides—especially NbC. 

The main problems identified in connection with the joining of nickel-based casting alloys, according to the strengthening type, are presented in Table 1 [17,30].

Despite numerous attempts to determine the HTBR and identify the hot-cracking criteria, mainly for wrought nickel-based alloys, there were no unambiguous research results that described cracking mechanisms in precipitation-strengthened nickel-based casting alloys and that evaluated and critiqued their weldability. 

The information available in the literature indicated that most nickel-based casting alloys, especially plastically deformed ones, belong to the weldable materials. However, nickel-based casting alloys, in particular those containing aluminum and titanium, are hard to weld, or even unweldable.

An example of a precipitation-strengthened nickel-based casting alloy is Inconel 713C, which is used for vital components of aircraft engines designed to operate at above 700 °C, such as turbine blades or vane clusters. 

The attempts at joining and repairing IN713C cast alloys by welding methods described in the literature to date have mainly concerned model components, whereas the translation of the technologies described into actual castings of complex shapes and various wall thicknesses has ended in failure, mainly due to hot cracking on the surface or inside the casting. 

Analysis of the literature data showed that due to its content of aluminum (approx. 6%) and titanium (up to 1%), Inconel 713C is classified as hard to weld or unweldable [31]. Thus, it is necessary to explore the mechanisms determining its hot-cracking susceptibility and to analyse the structural phenomena occurring during the crystallisation of remelted areas and padding welds in the casting repair process. 

The main purpose of conducted technological tests of remelting and pad welding for Inconel 713C precision castings and the performed remelting test under variable deformation conditions (transvarestraint test) was to assess the possibility of repairing or modifying the casting surface, and determine the criteria for hot remelting cracking. The determined range of technological parameters and hot-cracking criteria are the basis for the development of repair technology or even for the regeneration and modification of surface of Inconel 713C precision cast alloy. The performed structural tests presented an opportunity to describe HTBR and the mechanism of hot melt cracking for a remelted and pad-welded surface using the TIG method.

## 2. Materials and Methods

The material used in the tests was the nickel-based casting alloy Inconel 713C (New York, NY, United States), which is a polycrystalline, precipitation-strengthened material. The test material was delivered in the form of 5 mm thick plates and castings having a rectangular cross-section measuring 100 × 80 × 8 mm^3^. The test castings were made by precision casting. The vacuum induction melting (VIM) method was used to melt the charge material. 

The metallographic examinations were conducted using an Olympus GX71 (Warsaw, Poland) light microscope (LM) at magnifications of up to 500×. The surface structure after the tests was examined under scanning electron microscopes (SEM): a ZEISS Merlin Gemini II (Oberkochen, Germany) and a JEOL JCM-6000 Neoscope II (Tokyo, Japan). Images were recorded in the secondary electron mode at a magnification of 80,000× and at a voltage accelerating the electron beam to 15 keV.

**Figure 7:**
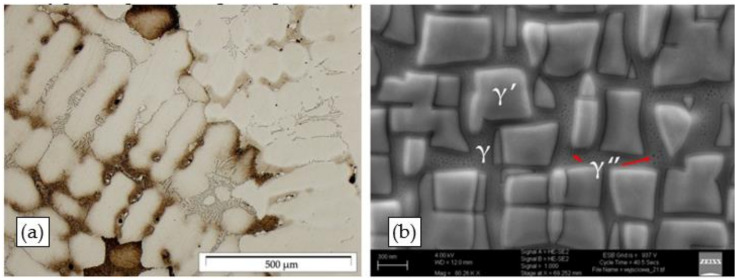
Structure of the Inconel 713C castings: (**a**) dendritic structure with visible eutectic mixtures and carbides (LM); (**b**) γ′ phase in the γ phase matrix (SEM).

The structural examinations of the Inconel 713C precision castings revealed that they had a dendritic structure (Figure 7a) with primary MC carbide precipitates (the main precipitate product of carbon) and eutectic mixture areas in interdendritic spaces (Figure 7b). The dendrites were built of the γ phase, being the matrix for γ′ phase precipitates. This is a typical structural arrangement for precision castings of IN713C, which was also confirmed by an analysis of the literature data [1,4,32]. The carbides observed were most frequently arranged in the “Chinese script” morphology.

Based on the literature data and a microanalysis of their chemical composition, it was confirmed that they were complex carbides containing Nb and Mo. Some fine-sized precipitates, which could be identified as the γ” phase, were also observed in the matrix. 

Subsequently, technological trials to repair simulated defects on the side surface of Inconel 713C precision castings were conducted using the TIG welding process. The TIG remelting and pad-welding tests were aimed at developing a technology for repairing surface defects in castings. The tests were performed using two gas shield variants: technically pure argon and a special gas mixture. 

The TIG remelting and pad welding in a pure argon atmosphere (99.995) by Messer (Bad Soden, Germany) was conducted using an Esab Aristotig 200 DC power supply (Gothenburg, Sweden), and a WT20 tungsten electrode by ESAB (Gothenburg, Sweden)with a diameter of 2.4 mm according to PN EN ISO 6848. The technological parameters of the processes are shown in Table 2. Thermanit 625 welding wire (EN ISO 18274–S Ni6625 (NiCr22Mo9Nb, AWS A5.14:ERniCrMo-3)) by Böhler Schweisstechnik GmbH (Linz, Austria), 1.0 mm in diameter, was used as filler material in the pad-welding tests. 

In the other test variant, the TIG remelting and pad-welding processes were conducted in the TecLine 8910 gas mixture by Messer (Bad Soden, Germany) (15% He, 2% H_2_, 0.015% N_2_, Ar-balance). A Lincoln Electric Bester Invertec V405-T Pulse power supply (Cleveland, OH, USA) and a tungsten electrode by ESAB (Gothenburg, Sweden) (WT20 according to the AWS classification), 2.4 mm in diameter, were used. The pad welding was performed using the same welding wire The pad welding was performed using the same welding wire by Böhler Schweisstechnik GmbH (Linz, Austria) (Thermanit 625, Ø1.0 mm) as in the case of the pad welding in an argon atmosphere. The parameters of the TIG remelting and pad-welding processes are set out in Table 3.

The influence of factors determining the viability of remelting of Inconel 713C was evaluated based on the results of the assessment of the HTBR under forced deformation conditions (transvarestraint test). The transvarestraint test consisted of fast bending of flat samples on a cylindrical die block, perpendicular to the direction of remelting [33]. The strain inflicted was related to the radius of the die block, and depended on the thickness of the bent specimen.

Cast plates of Inconel 713C measuring 100 × 80 × 5 mm^3^ were prepared for the tests. The remelting was performed with a direct current of 40 A, at a rate of approx. 1 mm/s. The remelting parameters were selected based on technological tests, so as to obtain full penetration. The strain inflicted in particular tests was calculated using the following Equation (1):

(1) $$\varepsilon =\frac{g}{2R}\cdot 100\%$$

where: ε—strain (%), g—specimen thickness (mm), and R—radius of die block curvature (mm) [34,35].

Following the remelting tests, the length of the longest crack in the remelted area axis (L_max_) and the total length of all cracks classified as hot cracks were determined. With the individual strain value during remelting (Equation (1)) and the welding rate (v_s_) being known, the crack growth time (t_max_) was calculated based on the following Equation (2):

(2) $$t_{\max}=\frac{L_{\max}}{v_{s}}$$

where: t_max_—crack growth time (s), L_max_—longest crack (mm), and v_s_—welding rate (mm/s) [34].

**Figure 8:**
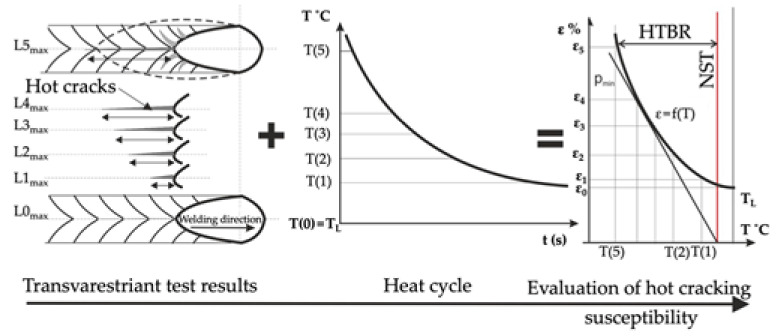
Methodology for determining the HTBR based on the results of the transvarestraint test.

With the welding heat cycle and the crack growth time during remelting being known, the temperature at the end of the longest crack was determined, which enabled identification of the HTBR for the Inconel 713C precision castings under variable strain conditions; i.e., under crystallisation conditions typical of welding processes. The schematic methodology is shown in Figure 8.

## 3. Results

**Figure 9:**
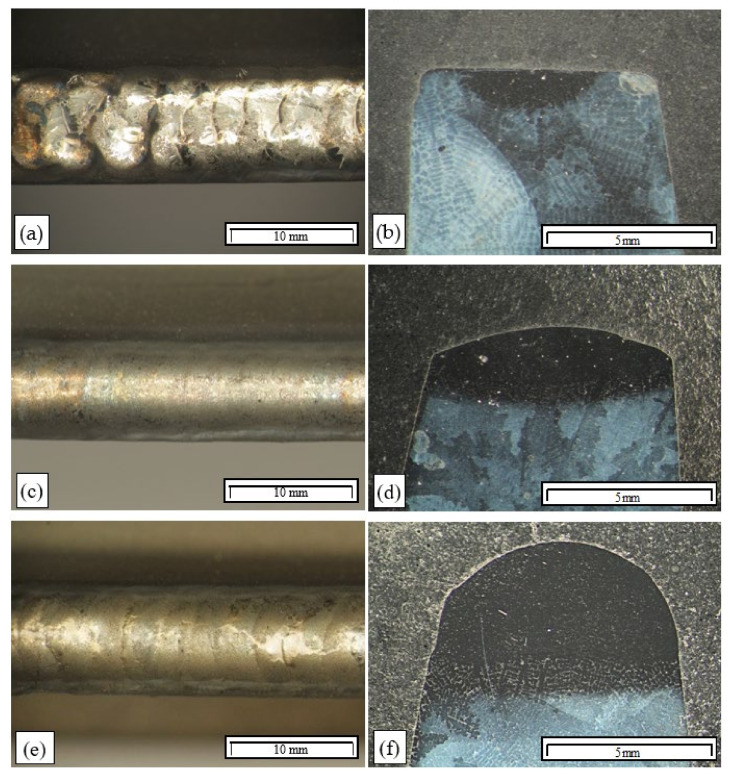
Surfaces and macrostructures of a remelted area and two padding welds made by TIG: (**a**,**b**) remelting of the base material with no filler, arc linear energy: 0.18 kJ/mm; (**c**,**d**) padding weld, arc linear energy: 0.34 kJ/mm; (**e**,**f**) padding weld made with Thermanit 625 wire, arc linear energy: 0.35 kJ/mm.

Visual examinations of the remelted area surfaces on the Inconel 713C precision castings obtained by TIG in an argon atmosphere revealed no cracks (Figure 9a,c,e). The surfaces obtained at an arc linear energy below 0.3 kJ/mm were uneven, with visible ripples (Figure 9a). Remelting at a higher linear energy (more than 0.3 kJ/mm) yielded an even and smooth surface (Figure 9c,e). Based on the visual examinations of the remelted area surfaces, they were classified as quality level C according to EN ISO 5817 (Table 2).

Visual examinations of the microstructure of the padding weld shown in Figure 9d revealed that the area of the padding weld material was built of narrow columnar dendrites that grew perpendicularly to the heat-dissipation direction. Partially melted dendrites of the base material were observed in the partially melted zone (Figure 9a,d,e). 

Examinations of the macrostructure of the remelted areas revealed that their width and depth increased with increasing arc energy (Figure 9b,d). Remelting at a linear energy of more than 0.3 kJ/mm resulted in the entire casting edge being remelted, which is important in the case of through-casting defects (Figure 9d). 

As for the pad welding performed with the use of Inconel 625 wire as the filler material, the padding-weld faces were correct (Figure 9e). They had a regular shape with no visible ripples on the surface. Pad welding with a linear energy of 0.3 kJ/mm resulted in the entire casting edge being remelted, and defects could be filled by filler material, depending on their size. Examinations showed that the padding welds had correct macrostructures. No cracks or other welding defects were identified in the padding welds or the HAZ. On this basis, the padding welds made at a linear energy of 0.35 kJ/mm could be classified as quality level B according to EN ISO 5817.

Examinations of the microstructure of the remelted areas obtained at a low linear energy (below 0.15 kJ/mm) confirmed that their surfaces were flat, with the weld lines being distinctly visible (Figure 9b). A broad partially melted zone was revealed (approx. 300 μm), in which the interdendritic zone was partially melted (Figure 9b).

**Figure 10:**
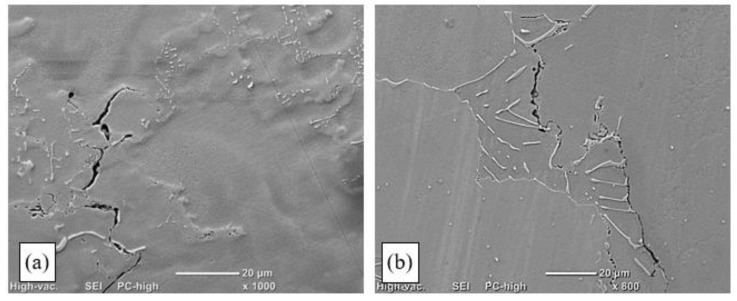
Structure of a remelted area on an Inconel 713C precision casting, obtained by TIG welding with no filler material (E_l_ = 0.38 kJ/mm): (**a**) crack in the partially melted zone; (**b**) cracks along dendrite boundaries in the area of “Chinese script” carbides.

In the case of the remelting process conducted at a linear energy of more than 0.21 kJ/mm, interdendritic cracks (Figure 10a) that disqualified the remelted areas for use were identified in the HAZ and the partially melted zone. Such cracks initiated along MC carbide boundaries in the partially melted zone. They formed as a consequence of the partial melting of dendrite branches and the loss of cohesion by the interdendritic liquid, which resulted in decreased adhesion to the base material.

**Figure 11:**
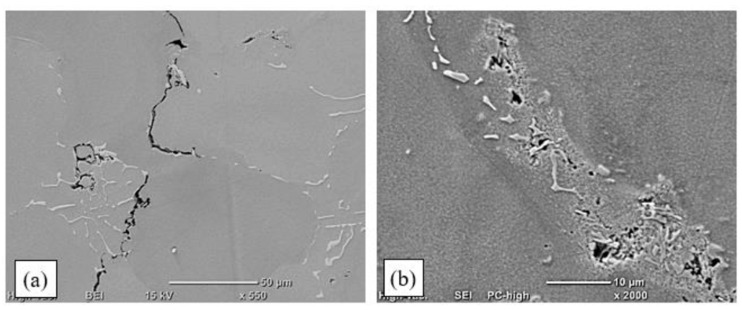
Structure of an Inconel 713C padding weld obtained by TIG welding with Inconel 625 as the filler material (E_l_ = 0.35 kJ/mm): (**a**) crack in the area of Chinese script carbide precipitates, SEM; (**b**) material discontinuities in the HAZ, in the area of the γ-γ′ eutectic mixture and carbides.

Dendrites were observed that had been separated from the base material and had not melted in the welding pool. This confirmed that deep penetration by liquid metal occurred in interdendritic spaces in the partially melted zone. The fragmentation of primary carbides was observed in those spaces, which was related to their partial melting and coagulation (Figure 11a). Numerous microcracks were also identified that ran along primary carbide precipitates, along dendrite boundaries (Figure 11b). Analysis of the crack trajectory confirmed that depending on the heat cycle of the pad-welding process, the cracks were related to the partial melting of dendrite edges (Figure 10a), eutectic mixture areas, and carbides (Figure 11b). Cracks initiated in the partially melted zone due to the rupture of the liquid film, which was stretched during padding-weld crystallisation [36,37]. 

**Figure 12:**
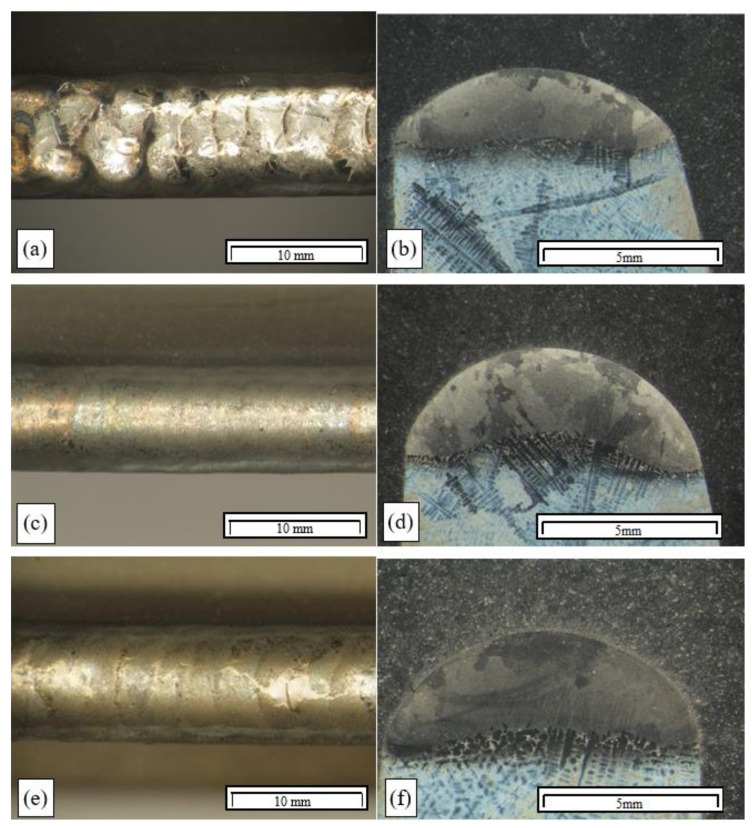
Surfaces and macrostructures of a remelted area and two padding welds made by TIG in a TecLine 8910 atmosphere: (**a**,**b**) remelting of the base material with no filler, arc linear energy: 0.15 kJ/mm; (**c**,**d**) padding weld, arc linear energy: 0.31 kJ/mm; (**e**,**f**) padding weld made with Thermanit 625 wire, arc linear energy: 0.35 kJ/mm.

**Figure 13:**
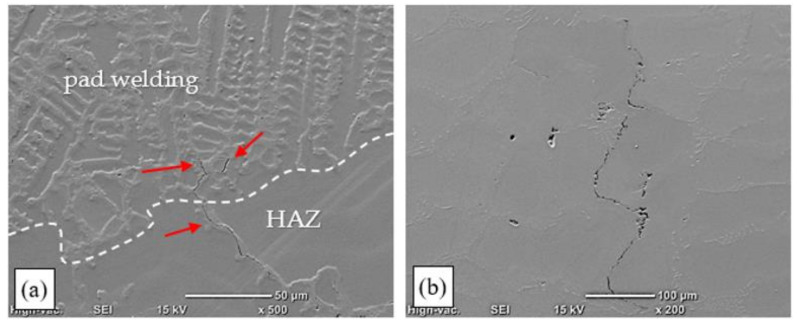
Structure of a remelted area in TIG remelting of Inconel 713C in a TecLine 8910 atmosphere (E_l_ = 0.35 kJ/mm): (**a**) crack along crystal boundaries in the HAZ; (**b**) crack along dendrite boundaries in the carbide area (SEM).

**Figure 14:**
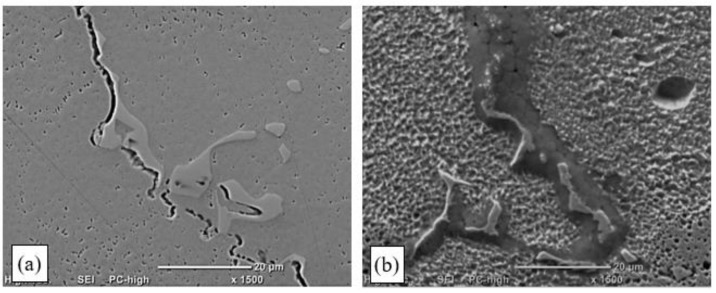
Padding-weld structure in TIG pad welding of Inconel 713C in a TecLine 8910 atmosphere (E_l_ = 0.35 kJ/mm) with filler material: (**a**) crack at a dendrite/carbide interface; (**b**) microcrack along dendrite boundaries with a visible privileged trajectory determined by carbides.

The use of the TecLine 8910 mixture increased the welding rate and improved the stability of electric arc discharges. An important technological measure affecting the remelting process was to increase molten metal liquidity by lowering the surface tension. This enabled filling developing cracks with liquid metal [16]. The process parameters are presented in Table 3, and examples of padding-weld faces and macrostructures are shown in Figure 12. Photographs of the microstructures of the remelted areas and padding welds obtained are shown in Figure 13 and Figure 14.

Visual examinations of the remelted area surfaces obtained by TIG remelting in a TecLine 8910 atmosphere revealed that in all cases, the surface was even and smooth, and free of welding defects (Figure 12a,c). Remelting with a linear energy of less than 0.17 kJ/mm led to the formation of ripples, caused by the gradual crystallisation of the molten pool (Figure 12a). Increasing the linear energy to more than 0.2 kJ/mm resulted in a smooth surface without visible ripples (Figure 12c). 

Examinations of the macrostructure revealed a correct remelted area geometry with distinctly marked zones; i.e., the melted metal, with visible dendrites growing in the heat-dissipation direction, a wide partially melted zone, and the HAZ. The remelting parameters applied enabled the melting of the entire casting edge (Figure 12b,d). Based on the visual examinations of the surfaces of the remelted areas and the assessment of their macrostructures, it was determined that the remelted areas met the requirements of quality level B according to EN ISO 5817.

Visual assessment of the padding-weld faces made by TIG in a TecLine 8910 atmosphere with the addition of Inconel 625 wire revealed that pad welding with a linear energy of up to 0.15 kJ/mm led to the formation of ripples on the surface. This was related to the feeding of filler material into the molten pool and the process of padding-weld crystallisation. Increasing the linear energy to more than 0.15 kJ/mm resulted in a smooth and even weld face (Figure 12e). Examinations of the padding-weld macrostructures revealed no welding defects. The shapes of the padding welds were found to be correct, with a clearly outlined fusion zone and an approx. 1 mm wide HAZ (Figure 12f). Examinations conducted in accordance with EN ISO 17637 enabled qualifying the padding welds as quality level B according to EN ISO 5817 (Figure 12e,f, Table 3). 

Analysis of the microstructure of the remelted areas obtained with a linear energy of less than 0.17 kJ/mm in a TecLine 8910 atmosphere revealed no cracks or other welding defects. A small number of hot cracks were only present in the HAZ of the remelted areas obtained with a linear energy of more than 0.17 kJ/mm. The cracks were found along dendrite boundaries, and their trajectories were determined by MC carbides (Figure 13).

The structure of the melted metal area was made up of fine columnar crystals, between which fine carbides, probably of the MC type, were revealed. In the fusion zone, the partial melting of dendrite boundaries was observed in the base material, as well as the partial melting of primary carbides, which had undergone fragmentation. On this basis, it can be stated that due to the identification in the interdendritic spaces of microcracks that were impossible to detect by nondestructive tests, this technology may be deemed acceptable, but is recommended only if the remelting is conducted with a linear energy of less than 0.17 kJ/mm.

The padding welds had a complex dendritic structure with carbides located in interdendritic spaces. This arrangement is typical of padding welds made on nickel-based casting alloys. The partial melting of carbides, leading to their coagulation and fragmentation, was also observed in the partially melted zone. The use of a gas mixture containing hydrogen and helium, increasing the arc linear energy and molten metal liquidity, resulted in a wider partially melted zone (approx. 300 μm), and thus enhanced the penetration of molten metal into interdendritic spaces.

In addition, in the case of the TIG pad welding in a TecLine 8910 atmosphere, microcracks were identified in the HAZ that had formed during pad welding at less than 0.17 kJ/mm. The cracks identified initiated at the weld line, where dendrites were partially melted. They grew as interdendritic cracks in the areas where MC primary carbides were present (Figure 14a).

The partial melting of carbides and dendrites was also observed in interdendritic spaces, which led—due to the ongoing crystallisation process—to the development of a network of fine material discontinuities that constituted DDC initiation spots (Figure 14b).

During the pad welding, similar to in the case of the remelting process, liquation cracks were identified in the HAZ. Although they were partially filled with metal, TIG pad welding should be deemed an acceptable technology only if the linear energy applied is below 0.17 kJ/mm, and if special production supervision and control conditions are satisfied. 

The measurements and calculations presented in Table 4 enabled the determination of the high-temperature brittleness threshold; i.e., the strain value at which no cracking occurred. The high-temperature brittleness threshold (ε_p_) adapted for the castings tested was 0.3%. This parameter can be adopted as a criterion for assessing the hot-cracking susceptibility of Inconel 713C. 

With the welding heat cycle and the crack growth time during remelting (Figure 8) being known, the temperature at the end of the longest crack was determined, which enabled the identification of the HTBR for the Inconel 713C precision castings under variable strain conditions; i.e., under crystallisation conditions typical of welding processes.

**Figure 15:**
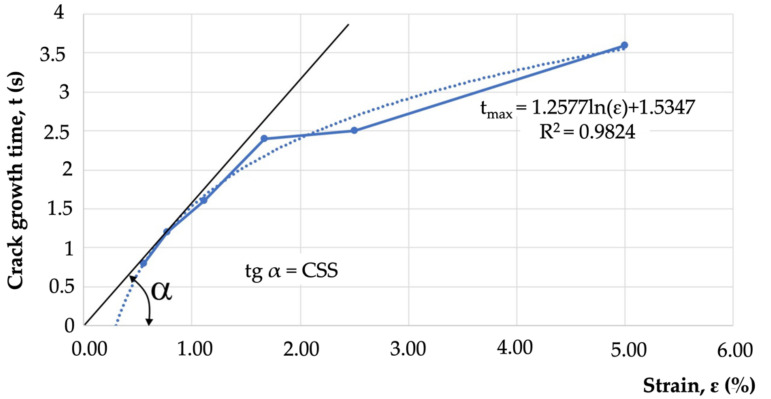
Hot-crack growth time as a function of specimen deformation in the transvarestraint test.The CSS value for the case in question was 1.71 1/s, which indicated that the alloy was highly susceptible to hot cracking during remelting. The results obtained enabled the determination of exponential ductility curves using ε = f(T) (Figure 16).

Determination of the relation of t_max_ = f(ε) also enabled the determination of the value of the critical strain speed (CSS) parameter, understood as the tangent of the inclination angle between the tangent to the crack growth curve and the deformation axis (Figure 15). 

The results of the tests enabled the determination of the maximum crack length in the padding-weld axis (L_max_), the total crack length (L1_max_), the cracking threshold (ε_p_), the HTBR during welding, the critical strain rate for temperature drop (CST), and the critical strain speed (CSS). The results obtained made it possible to describe the phenomena occurring during padding-weld crystallisation and the factors affecting hot-cracking susceptibility within the HTBR, and thus to assess the weldability of Inconel 713C and the possibility of repairing defects in Inconel 713C castings.

**Figure 16:**
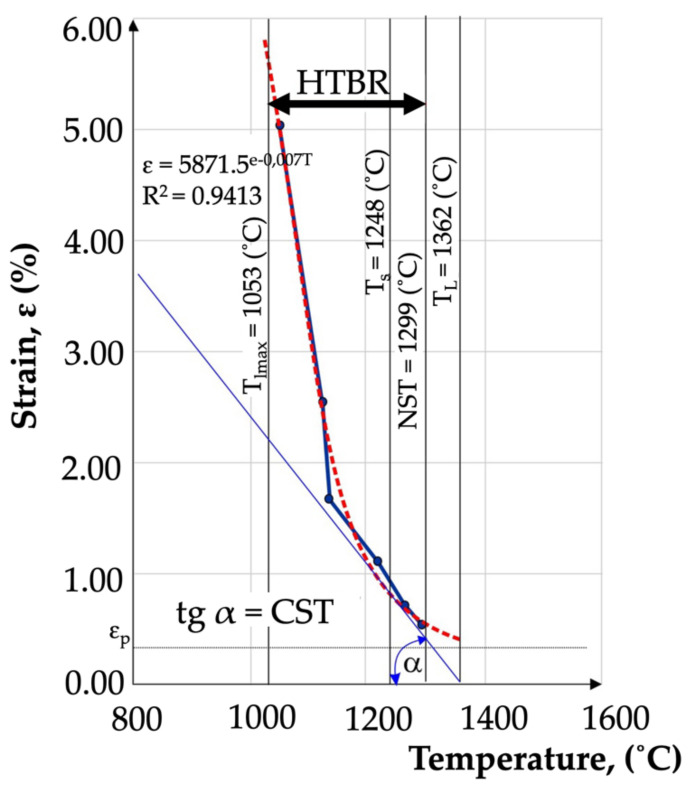
Strain as a function of temperature for Inconel 713C precision castings, determined based on transvarestraint tests.

Based on the regression and correlation analysis of a single variable function (nonlinear), it was found that the relationship determined was valid. The relation described enabled the determination of the HTBR under remelting conditions. The HTBR is defined as the difference between the NST and the temperature at the end of the longest crack. The relation also enabled the determination of certain hot-cracking criteria, including the critical strain rate for temperature drop (CST), which is the tangent of the angle between the tangent to the ductility curve ε = f(T) and the temperature axis (Figure 16). The value of this parameter was 0.0055 1/°C.

**Figure 17:**
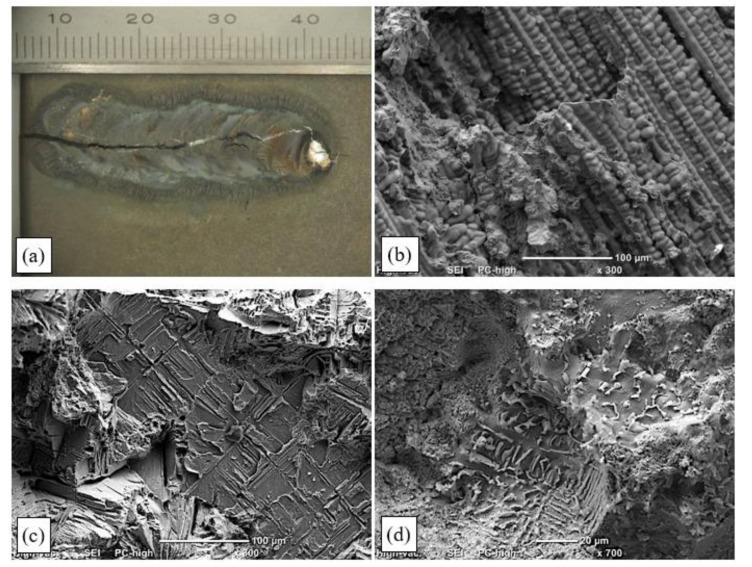
Results of fractographic examinations of the surface of a hot crack that developed during a transvarestraint test in a specimen subjected to 5% strain during remelting: (**a**) general view; (**b**) ruptured dendrites and interdendritic bridges in the remelted area; (**c**) crack surface with visible brittle transcrystalline fracture and areas of liquid film rupture; (**d**) partially melted carbides in the partially melted zone.

Figure 17a shows the weld face on a specimen that was subjected to maximum deformation during the transvarestraint test (ε = 5%). It was found that the hot crack caused by specimen deformation ran along the axis of the padding weld and across its entire melted part, which indicated its brittleness. Fractographic examinations confirmed that fine columnar dendrites grew perpendicularly to the remelted area surface, in the heat-dissipation direction.

The crack initiation site was the molten pool, where the interdendritic liquid film lost cohesion at the NST due to tensile stresses involved in the crystallisation process. The rupture of “bridges” that formed the rigid structure of the liquid–solid state was also observed there (Figure 17b). The number of bridges was relatively small, and the dominant crack-initiation mechanism was the loss of continuity by the liquid film covering the crystallising dendrites. As the temperature dropped, the solid body lattice expanded, and thus the number of ruptured bridges between dendrite branches increased (Figure 17b). Near the solidus temperature, the inflow of liquid metal into the crystallising area of the padding weld stopped, leading to the formation of local voids, which—with the material’s ductility dropping in the HTBR—reinforced the tendency for cracks to propagate (Figure 17c). Partially melted interdendritic spaces with distinctly visible carbides were observed in the partially melted zone (Figure 17d).

Brittle transcrystalline fracture surfaces were also observed (Figure 17d). They were ruptured base material dendrites that were partially melted. The fractographic examinations of hot-crack surfaces confirmed the same hot-cracking mechanism for all cases (irrespective of the strain degree).

## 4. Discussion

The analysis of the results of the technological TIG remelting and pad-welding tests in an argon atmosphere showed that the process could not be used for repairing precision castings. Despite correct surfaces having been obtained (particularly in the pad-welding process) (Figure 9), the examinations of the microstructure revealed numerous cracks in the heat-affected zone and the partially melted zone (Figure 10 and Figure 11). The areas that were the most susceptible to hot cracking were the interdendritic spaces of the base material that underwent partial melting. As a result of the plastic strains at work, the liquid metal lost cohesion. It was found that the areas privileged for the appearance of cracks were sites with carbides in the Chinese script morphology (Figure 10b and Figure 11a).

In order to enhance electric arc stability and increase metal liquidity during the TIG welding tests, some of the tests were conducted with a new gas mixture—TecLine 8910, containing approx. 15% He and 2% H_2_. The gas mixture considerably improved the quality of the surfaces obtained (Figure 12); however, correct results were only obtained for remelted areas and padding welds made with a linear energy of less than 0.17 kJ/cm. Remelting and pad welding at a higher energy resulted in the formation of interdendritic cracks, which was related to strain occurring in the HAZ and resulting from the welding heat cycle (Figure 13).

The hot cracks revealed on metallographic specimens were most often located under the remelted and pad-welded surface, which made it impossible to identify defects by nondestructive tests. However, due to the need to ensure the safe use of the repaired elements, it was necessary to perform RTG examinations of each repaired casting. Further investigation of the mechanical properties is also advisable for repaired castings, especially in the field of creep resistance. Such requirements should be included in the qualification procedure for Inconel 713C precision-casting repair technology.

The high-temperature brittleness range (HTBR) determined in the transvarestraint test was understood as the difference between the longest crack temperature and the NST. The range had a width of 246 °C, and extended from 1053 °C to 1299 °C (Table 4). It was found that the HTBR under remelting conditions was nearly 5 times wider than the HTBR determined for the base material [38]. This indicated that the material was much more susceptible to cracking in a remelting process involving concentrated arc energy than under conditions of even heat distribution involved in Gleeble 3500 simulations [38]. 

The level of plastic strain at which no cracking occurred in a casting under remelting conditions was 0.3%. This was the high-temperature brittleness threshold, or the so-called reserve of plasticity described in Prokhorov’s theory [20].

The results of the transvarestraint tests also enabled the determination of crystallisation cracking criteria. The critical strain speed (CSS), which for IN713C was 1.71 (1/s) (Table 4), was used as the criterion for the strain rate during remelting. If this CSS value was exceeded, crystallisation cracks would appear in the material during remelting (Figure 14). Another indicator describing the cracking susceptibility of a casting during remelting is the critical strain rate for temperature drop (CST), which for IN713C was 0.0055 1/°C. If this value was exceeded, cracking occurred. The schematic value of the CST, defined as the tangent of the angle between the tangent to the ductility curve and the temperature axis, is shown in Figure 15. If angle α was wider than the critical angle, the material cracked.

**Figure 18:**
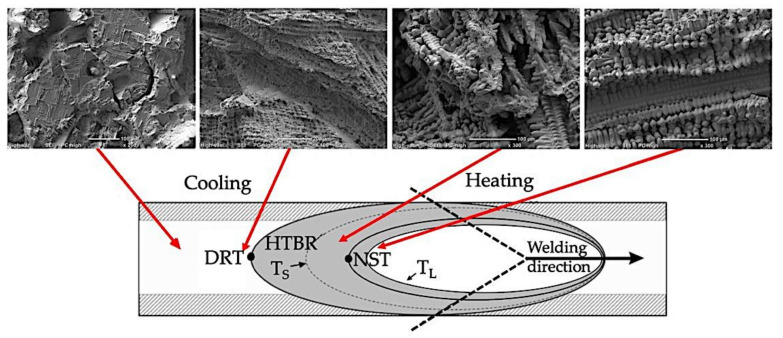
Change in surface morphology on the surface of a crystallisation crack that developed during the remelting of a casting under forced deformation conditions. T_Lmax_—temperature at the end of the longest crack.

Examinations of the surfaces of the crystallisation cracks that appeared during the deformation of the remelted specimens in the transvarestraint tests indicated a similar cracking mechanism to the case of specimens deformed using a Gleeble simulator. An area was observed on the crack surface where parallel dendrites developed. It was found that the fracture surface changed within the area where columnar dendrites were present (i.e., within the melted metal area) (Figure 17c). An area typical of cracking (close to the NST) was also identified, where ruptured bridges and dendrites in the liquid–solid state were present (Figure 17b). Below the solidus lines, brittle fracture surfaces were observed in which voids had formed in the liquid–solid state due to the partial melting of dendrite edges and carbides (Figure 17d). A schematic change in the fracture surface structure is shown in Figure 18.

The obtained results of the technological and structural tests, including the description of the hot-cracking mechanism in HTBR and the determination of numerical fracture criteria in the form of indicators (ε_p_, CST, and CSS), constituted a unique contribution to the understanding of the weldability of the Inconel 713C alloy. They are also a background to the evaluation of the possibility of using welding techniques for repair, regeneration, or surface modification of precision castings composed of Inconel 713C alloy.

The hot-cracking criteria and mechanisms described were used to devise technological tests for remelting and pad welding of Inconel 713C precision castings. Based on the results obtained and the requirements set by manufacturers and users, a number of welding technologies were selected that had the greatest potential for use in the repair of aircraft-engine components. 

## 5. Conclusions

The test results presented confirmed the hypothesis that the possibility of repairing Inconel 713C precision castings is decided by hot-cracking susceptibility, which is the effect of structural phenomena occurring during padding-weld crystallisation. Based on their analysis, the following conclusions were formulated:

1. The critical strain speed (CSS) of 1.71 1/s and the critical strain rate for temperature drop (CST), in this case having the value of 0.0055 1/°C, should be adopted as the criteria for assessing the hot-cracking susceptibility of Inconel 713C within the high-temperature brittleness range.
2. Hot cracks appearing when the alloy was being remelted under forced deformation conditions developed within the high-temperature brittleness range. This was caused by voids, the formation of which was related to the loss of cohesion by the interdendritic liquid and the rupture of the solid body lattice formed of columnar dendrites. Areas with carbides in the Chinese script morphology favoured the development of hot cracks.
3. Hot cracks in the HAZ and the partially melted zone resulted from the critical strain being exceeded during the crystallisation of remelted areas or padding welds. The Inconel 713C alloy was susceptible to cracking during plastic deformation in the HAZ at temperatures above 1050 °C. The critical circumferential strain for this temperature was 0.48%.
4. The main difficulty in repairing Inconel 713C castings, as identified during the technological TIG tests, was due to microcracks initiating in the partially melted zone and propagating into the HAZ. Due to their size and location, such cracks were very difficult to detect by nondestructive testing methods.
5. Under variable-strain conditions characteristic of the remelting and pad-welding processes, the high-temperature brittleness range widened nearly 5-fold (the HTBR width was 246 °C), and extended from 1053 °C to 1299 °C. The strain below which the material was resistant to hot cracking was 0.3%.

## Figures and Tables

**Table 1:** The parameters of main weldability concerns for nickel-based alloys [17].

Material Type	Strengthening Type	Main Components	Examples of Alloys	Main Weldability Concerns
**Heatproof**	**Solution Strengthened**	Ni–Cu	Monel 400, Monel K-500 (New York, NY, USA)	Weld porosity, crystallisation cracking
		Ni–Mo	Hastelloy B-2 (Kokomo, IN, USA)	Weld and HAZ corrosion
		Ni–Cr–Mo	Hastelloy G-35 (Kokomo, IN, USA)	Weld and HAZ corrosion
		Ni–Cr–Mo–W	Hastelloy C-22 (Kokomo, IN, USA), Inconel 686 (New York, NY, USA)	Weld and HAZ corrosion
		Ni–Cr–Mo–Cu	Hastelloy C-2000 (Kokomo, IN, USA)	Weld and HAZ corrosion
**Creep-resistant**	**Solution Strengthened**	Ni–Fe–Cr	Incoloy 800H (New York, NY, USA), RA330 (Temperance, MI, USA)	Liquation cracking
		Ni–Cr–Fe	Inconel 600, Inconel 690 (New York, NY, USA)	DDC
		Ni–Cr–Fe–Mo	Hastelloy X (Kokomo, IN, USA)	Liquation cracking
		Ni–Cr–Mo–Nb	Inconel 625 (New York, NY, USA), Haynes 625SQ (Kokomo, IN, USA)	Crystallisation cracking
		Ni–Cr–Co–Mo	Inconel 617 (New York, NY, USA)	Liquation cracking
		Ni–Cr–W–Mo	Haynes 230 (Kokomo, IN, USA)	Crystallisation and liquation cracking
		Ni–Co–Cr–Si	Haynes R-160 (Kokomo, IN, USA)	Crystallisation cracking
	**Precipitation-Strengthened**	γ′ phase	Rene 41 (Boston, MA, USA), Waspaloy (Hartford, CT, USA), Inconel 713C (New York, NY, USA)	Annealing, crystallisation, and liquation cracking
		γ″ phase	Allvac 718Plus (Pittsburgh, PN, USA)	Crystallisation and liquation cracking
		Ni_3_Al	IC-218, IC-25 (Ohio, OH, USA)	Crystallisation and liquation cracking
	**Dispersion Strengthened**	Y_2_O_3_	Inconel MA754, Inconel MA6000 (New York, NY, USA)	Metal oxidation

**Table 2:** Parameters of the Tungsten Inert Gas remelting and pad-welding processes in an argon atmosphere.

Specimen Designation		Current (A)	Arc Voltage (V)	Remelting /Pad-Welding Rate (mm/s)	Arc Linear Energy (kJ/mm)	Gas Flow Rate (l/min)	Visual Assessment of the Weld Face According to EN ISO 5817
**Remelting**	15	25	12	1.20	0.15	12	C
	16	30	12	1.20	0.18	12	C
	17	35	12	1.20	0.21	12	C
	18	40	15	1.20	0.30	12	C
	19	45	15	1.20	0.34	12	C
	20	50	15	1.20	0.38	12	B
**Pad Welding**	625.1	30	15	1.03	0.26	7	B
	625.2	35	15	1.03	0.31	7	C
	625.3	40	15	1.03	0.35	12	B

**Table 3:** Parameters of the TIG remelting and pad-welding processes in a TecLine 8910 gas mixture atmosphere.

Specimen Designation		Current (A)	Arc Voltage (V)	Remelting/Pad-Welding Rate (mm/s)	Arc Linear Energy (kJ/mm)	Gas Flow Rate (l/min)	Visual Assessment of the Weld Face According to EN ISO 5817
**Remelting**	1	25	12	1.30	0.15	12	B
	2	30	12	1.30	0.17	12	B
	3	35	12	1.30	0.19	12	B
	4	40	15	1.30	0.28	12	B
	5	45	15	1.30	0.31	12	B
	6	50	15	1.30	0.35	12	B
**Pad Welding**	7	25	10	1.15	0.13	7	B
	8	30	12	1.15	0.17	7	B
	9	35	12	1.15	0.22	12	B
	10	40	15	1.15	0.31	12	B
	11	45	15	1.15	0.35	12	B
	12	50	15	1.15	0.39	12	B

**Table 4:** Results of the measurements and calculations of the indicators used to assess the high-temperature brittleness range of the Inconel 713C precision castings.

No.	Strain ε (%)	Longest Crack Length L_max_ (mm)	Crack Growth Time t_max_ (s)	Critical Strain Speed (1/s)	Critical Strain Temperature (1/°C)	ΔHTBR ** (°C)	HTBR ** (°C)
1	0.56	4	0.8	1.71	0.0055	1053–1299	246
2	0.77	6	1.2				
3	1.12	8	1.6				
4	1.67	12	2.4				
5	2.50	12.5	2.5				
6	5.00	18	3.6				

** The results are presented in [38].

## Data Availability

The data supporting reported results are not stored in any publicly archived datasets. Readers can contact the corresponding author for any further clarification of the results obtained.
